# Mechanisms for the control of local tissue blood flow during thermal interventions: influence of temperature‐dependent ATP release from human blood and endothelial cells

**DOI:** 10.1113/EP085910

**Published:** 2017-02-01

**Authors:** Kameljit K. Kalsi, Scott T. Chiesa, Steven J. Trangmar, Leena Ali, Makrand D. Lotlikar, José González‐Alonso

**Affiliations:** ^1^ Centre for Human Performance, Exercise and RehabilitationBrunel University LondonUxbridgeUK; ^2^ Department of AnaestheticsEaling Hospital NHS TrustSouthallUK

## Abstract

**New Findings:**

- **What is the central question of this study?**
  Skin and muscle blood flow increases with heating and decreases with cooling, but the temperature‐sensitive mechanisms underlying these responses are not fully elucidated.
- **What is the main finding and its importance?**
  We found that local tissue hyperaemia was related to elevations in ATP release from erythrocytes. Increasing intravascular ATP augmented skin and tissue perfusion to levels equal or above thermal hyperaemia. ATP release from isolated erythrocytes was altered by heating and cooling. Our findings suggest that erythrocytes are involved in thermal regulation of blood flow via modulation of ATP release.

Local tissue perfusion changes with alterations in temperature during heating and cooling, but the thermosensitivity of the vascular ATP signalling mechanisms for control of blood flow during thermal interventions remains unknown. Here, we tested the hypotheses that the release of the vasodilator mediator ATP from human erythrocytes, but not from endothelial cells or other blood constituents, is sensitive to both increases and reductions in temperature and that increasing intravascular ATP availability with ATP infusion would potentiate thermal hyperaemia in limb tissues. We first measured blood temperature, brachial artery blood flow and plasma [ATP] during passive arm heating and cooling in healthy men and found that they increased by 3.0 ± 1.2°C, 105 ± 25 ml min^−1^ °C^−1^ and twofold, respectively, (all *P* < 0.05) with heating, but decreased or remained unchanged with cooling. In additional men, infusion of ATP into the brachial artery increased skin and deep tissue perfusion to levels equal or above thermal hyperaemia. In isolated erythrocyte samples exposed to different temperatures, ATP release increased 1.9‐fold from 33 to 39°C (*P* < 0.05) and declined by ∼50% at 20°C (*P* < 0.05), but no changes were observed in cultured human endothelial cells, plasma or serum samples. In conclusion, increases in plasma [ATP] and skin and deep tissue perfusion with limb heating are associated with elevations in ATP release from erythrocytes, but not from endothelial cells or other blood constituents. Erythrocyte ATP release is also sensitive to temperature reductions, suggesting that erythrocytes may function as thermal sensors and ATP signalling generators for control of tissue perfusion during thermal interventions.

## Introduction

Localized heating and cooling have long been known to alter skin perfusion in resting human limbs, such that blood flow increases with heating and declines with cooling (Barcroft & Edholm, 1943). It is now recognized that the global differences in limb perfusion evoked by heating and cooling encompass macro‐ and microcirculatory changes in multiple limb tissues, including skeletal muscle, fat, bone and skin (Heinonen *et al*. 2011; Pearson *et al*. 2011). In support of the responsiveness of skeletal muscle macro‐ and microcirculations to thermal stimuli, local cooling and heating have been shown to change perfusion in the human thenar eminence muscle group in proportion to variations in intramuscular temperature (Binzoni *et al*. 2012), an observation which is consistent with the local responses seen in porcine skeletal muscle with microwave heating (Akyürekli *et al*. 1997), and the responses to different thermal interventions of the human lower leg muscles (Heinonen *et al*. 2011) and the profunda femoral artery, which primarily supplies blood to the thigh muscles (Chiesa *et al*. 2015, 2016). These haemodynamic responses and their impact on the supply of regulatory substances, oxygen and substrates form the basis for the widespread clinical and ergogenic application of heat and cold therapies and thermal interventions (Imamura *et al*. 2001; Malanga *et al*. 2015). However, the vascular signalling mechanisms involved in the regulation of limb tissue perfusion with changes in local temperature are as yet not fully characterized or understood.

Heat stress‐induced tissue hyperthermia, in isolation or combined with exercise, is associated with increases in intravascular ATP and accompanied by vasodilatation of the human skin and deep limb tissue vasculatures (Pearson *et al*. 2011). This supports a coupling between the increase in intravascular ATP and the concomitant elevation in local perfusion (Ellsworth *et al*. 1995; Kalsi & González‐Alonso, 2012). However, the ATP and blood flow responses to isolated limb temperature manipulations, including cooling, are unknown. The argument that reduced intravascular ATP might be associated with lower tissue perfusion has been made in the literature with respect to the tissue hypoperfusion seen with ageing (Kirby *et al*. 2012). One of the main observations with exposure to heat stress is that blood flow in the skin is enhanced and can be increased by many‐fold in the forearm with local heating (Pergola *et al*. 1993), without an accumulation of ATP in the interstitial space of human skin (Gifford *et al*. 2012). However, the involvement of intravascular ATP in regulating skin blood flow during heat stress has never been investigated. Therefore, one of the aims of the present study was to determine whether increasing intravascular ATP availability with infusion of ATP in graded doses in the brachial artery would enhance skin and arm blood flow to levels seen during thermal hyperaemia and whether the vasodilator response to graded ATP infusion is potentiated or remains the same following local arm heating.

In *in vitro* conditions, we have demonstrated that erythrocytes are the only blood source of ATP when isolated red blood cells, plasma or serum are heated from 33 to 42°C (Kalsi & González‐Alonso, 2012). This temperature‐dependent ATP release from human erythrocytes is blocked by administration of inhibitors of cystic fibrosis transmembrane regulator (a regulator of ATP‐releasing channels), suggesting that ATP release is a transport‐mediated process (Kalsi & González‐Alonso, 2012). The temperature‐sensitive mechanism of erythrocyte ATP release might be different from mechanisms responsive to changes in blood oxygen, shear stress, pH and lactic acid normally occurring in metabolically active tissues, such as contracting skeletal muscle (Bodin & Burnstock, 1995; Ellsworth *et al*. 1995; González‐Alonso *et al*. 2002, 2015; Gourine *et al*. 2010), because metabolic changes in response to local heating and cooling are relatively small (Barcroft & Edholm, 1943; Pearson *et al*. 2011). It remains unknown, however, whether reductions in temperature depress ATP release from isolated human erythrocytes. Moreover, no study to date has examined the relationship between blood temperature, plasma [ATP] and limb tissue blood flow during local heating and cooling in resting individuals and compared the responses with those from *in vitro* studies assessing the impact of larger physiological variations in temperature on ATP release from different blood constituents. It is also equally possible that the released ATP derives from the endothelium, because shear stress, hypoxia and intracellular Ca^2+^ agonists have all proved to be effective in stimulating the release of ATP from endothelial cells (Bodin & Burnstock, 1995; Gödecke *et al*. 2012). It is unknown, however, whether a temperature‐sensitive ATP release mechanism is also present in endothelial cells.

Therefore, the aim of the study was fourfold: (i) to investigate the relationships among local arm haemodynamic responses, local blood temperature (*T*_b_) and plasma [ATP] during isolated arm heating and cooling; (ii) to determine whether intrabrachial artery infusion of ATP can potentiate the heating‐mediated increases in skin and deep tissue blood flow in the human arm; (iii) to characterize the influence of physiological temperature variations on ATP release from isolated erythrocytes, plasma and serum; and (iv) to investigate whether human endothelial cells are another source of extracellular ATP during changes in temperature. We hypothesized that changes in limb skin and tissue blood flow with heating and cooling would be closely related to changes in blood temperature and plasma [ATP] *in vivo* and that ATP release from erythrocytes, but not from plasma, serum or endothelial cells, would be sensitive to both increases and decreases in temperature. Furthermore, increases in intravascular ATP availability with graded ATP infusion will elevate both cutaneous and skeletal muscle blood flow to levels above those seen with thermal hyperaemia, a response that would be enhanced further when ATP is infused in the heated arm.

## Methods

### Subjects

This study consisted of four experimental protocols (i.e. two *in vivo* protocols in healthy human subjects and two *in vitro* protocols using human blood and endothelial cells). The study conformed to the code of ethics of the World Medical Association (*Declaration of Helsinki*) and was conducted after ethical approval from the Brunel University London Research Ethics Committee. Informed written and verbal consent was obtained from all the participants before commencing any part of this study. All participants were asked to refrain from exercise and ingestion of caffeine before all experiments. Different subsets of participants were recruited for each experimental protocol. In total, 18 healthy men participated in the first (mean ± SD: age 21 ± 2 years, height 175 ± 6 cm and weight 74 ± 6 kg; *n* = 10) and the second *in vivo* protocols (age 22 ± 4 years, height 177 ± 8 cm and weight 75 ± 6 kg; *n* = 8). For the subsequent *in vitro* protocols, blood constituents were separated from venous blood samples taken from a different subset of 12 healthy men ranging in age from 21 to 46 years (mean ± SD: age 28 ± 7 years), whereas endothelial cells were obtained from three different healthy human umbilical cords.

### Protocol 1: effects of heating and cooling on blood temperature, plasma ATP and limb haemodynamics *in vivo*

Throughout the experimental protocol, subjects remained in the supine position with both arms resting at an 80 deg angle to the torso. Baseline measurements were taken 30 min after insertion of catheters and placement of monitoring equipment. The order of heating and cooling protocols was counterbalanced across subjects. A custom‐built tube‐lined cuff was placed on the experimental arm and perfused with water at 49°C in order to allow heating of the isolated limb. Flow measurements and blood samples for plasma ATP and blood gases from both arms were taken at 10 min intervals for the duration of 60 min of heating, with the control arm remaining uncovered at an ambient temperature of 20–22°C. Before starting the second part of the study, the participants were allowed to rest for an hour while skin and blood temperatures returned to baseline. For the cooling protocol, the forearm of the experimental arm was covered with a tight‐fitting plastic bag and encased in crushed ice placed in a custom‐built polystyrene box, with the upper arm proximal to the catheters simultaneously cooled with crushed‐ice packs. The hand and the wrist area were exposed to the cold temperature reached within the box (∼13°C), but were not covered with ice in order to avoid the cold pressor effect, which could potentially affect both blood pressure and heart rate (Mourot *et al*. 2009). Ice was kept on the arm for 60 min, and measurements were taken every 10 min.

#### Arterial and venous catheterizaion

Prior to commencement of the first experimental protocol, an 18 gauge, 4.5 cm catheter was inserted in a retrograde direction into an antecubital vein of both the experimental and the control arms for obtaining deep venous blood samples (Mottram, 1955). This line was continuously flushed with sterile saline. A second catheter was placed in both arms within ∼1 cm of the first one for the insertion of a sterile implantable thermocouple microprobe (T‐204F; Physitemp, Clifton, NJ, USA) to measure *T*_b_ continuously. The second catheter was carefully removed, while the thermocouple remained 3–4 cm deep in the vein and secured in place with micropore tape.

#### Temperature measurements

For the first protocol, skin temperature (*T*_sk_) was assessed using skin thermistors placed on two sites on each arm (type‐t thermocouples; Grant Instruments, Cambridge, UK) and securely held in place throughout the protocol by the use of medical tape. Core temperature (*T*_c_) was assessed using a commercially available rectal probe (Thermalert; Physitemp), inserted 10 cm past the sphincter muscle using a commercially available rectal probe (Thermalert; Physitemp). Skin, rectal and venous thermistors were connected to two four‐channel amplifiers (TC‐2000; Sable Systems, Las Vegas, NV, USA) and a data acquisition system (Powerlab 16/30 ML 880/P; ADInstruments, Bella Vista, NSW, Australia).

#### Haemodynamic measurements

Brachial artery blood flow (BABF) was examined with the arm extended and positioned at an angle of ∼80 deg from the torso. Flow was measured using an ultrasound Doppler machine equipped with a 10 MHz linear probe (Vivid 7 Dimension; GE Medical, Horton, Norway) and was calculated using the following equation:

Brachial  artery  blood  flow ( ml  mi n−1)=V mean ×Π×D/22×60

where *V*_mean_ is time‐averaged mean blood velocity (in centimetres per second), Pi and *D* is the vessel diameter (in centimetres). Brachial artery vessel diameter was determined at peak systole from three two‐dimensional images in the longitudinal view at ∼70 frames s^−1^ (Chiesa *et al*. 2015). Mean blood velocity was measured using continuous pulsed‐wave Doppler at a frequency of 4.4 MHz, with an insonation angle consistently below 60 deg and the sample volume extended to cover the entire vessel lumen. Blood velocity was calculated from the average of three measurements, each consisting of 10–12 velocity profiles. Blood pressure was measured non‐invasively by finger photoplethysmography (Finometer, FMS, Amsterdam, The Netherlands) via a cuff on the middle finger of the control hand. Cardiac output was calculated as stroke volume multiplied by heart rate, with stroke volume being estimated using the ModelFlow method (Wesseling *et al*. 1993; Beatscope, FMS, The Netherlands) and heart rate determined using a three‐lead ECG. Arm and systemic vascular conductance were calculated as the brachial arterial blood flow/mean arterial pressure and cardiac output/mean arterial pressure, respectively. Skin blood flow was measured via laser‐Doppler flowmetry (Periflux™ Flowmetry System, Järfälla, Sweden). One probe was secured to the ventral surface of the forearm (i.e. above the flexor carpi radialis) of the control arm and another on the experimental arm. Tissue oxygenation in and surrounding the main muscle of the forearm (extensor carpi ulnaris) was measured using near‐infrared spectroscopy (NIRS; INVOS Cerebral Oximeter; Somanetics, Troy, MI, USA). The optode pads were placed on the skin surrounding this muscle of both the control and experimental arm and taped to reduce interference from external light sources. In the ATP infusion protocol, the NIRS optode was placed on the forearm, thus below the point of ATP infusion into the brachial artery.

#### Blood sampling for ATP and blood gases

Blood samples for ATP analysis for the *in vivo* protocol were collected directly in monovette syringes (S‐monovette, 2.7 ml KE; Sarstedt, Nümbrecht, Germany) containing stop solution (1.7 ml), which was prepared according to previously described methods (Gorman *et al*. 2003). Briefly, the stop solution contained 5 nmol l^−1^ *S*‐(4‐nitrobenzyl)‐6‐thioinosine, 100 μmol l^−1^ 3‐isobutyl‐1‐methylxanthine, 10 μmol l^−1^ forskolin, 4.15 mmol l^−1^ EDTA, 118 mmol l^−1^ NaCl, 5 mmol l^−1^ KCl and 40 mmol l^−1^ tricine buffer. Samples were weighed and immediately centrifuged for 1 min at 15,493*g*. The use of stop solution prevents any further release and metabolism of ATP and remains stable for ∼30 min (Gorman *et al*. 2003, 2007). ATP was determined with the luciferin–luciferase technique, using a luminometer with three automatic injectors (Orion Microplate Luminometer; Berthold Detection System GmbH, Pforzheim, Germany). ATP in the supernatant was measured in duplicates at room temperature (20–22°C) using an ATP kit (ATP Kit SL; BioThema AB, Dalarö, Sweden) with an internal ATP standard procedure. Haemoglobin concentration ([Hb]) was measured to estimate the degree of haemolysis by reading the absorbance of the supernatants at 560, 577 and 593 nm (Cripps, 1968) using a spectrophotometer (Jenway 3500, Felsted, UK). To account for ATP from red blood cell (RBC) haemolysis, a correction curve was set up as outlined previously (Gorman *et al*. 2007). The linear regression line of lysed RBC [ATP] (in nanomoles per litre) was plotted against the [Hb] (in milligrams per decilitre), with the relationship *y* = 4.61*x* − 4.79 and *R* ^2^ = 0.99. Any samples that were >2SD from the mean percentage haemolysis were excluded and considered as experimental error (<5% were excluded). To measure blood gas variables, haemoglobin, glucose and lactate concentrations, 1 ml of blood was drawn into preheparinized syringes for blood gas sampling (PICO syringes; Radiometer, Copenhagen, Denmark). Blood gas parameters were measured using an automated analyser (ABL 825 M Flex; Radiometer, Copenhagen, Denmark). Forearm oxygen consumption (${\dot{V}}_{O_{2}}$) was estimated in the experimental and control arms using the Fick equation (${\dot{V}}_{O_{2}}$ = BABF × arteriovenous O_2_ difference), where arterial O_2_ content was calculated assuming that the arterial [Hb] was the same as the measured venous [Hb] and that arterial O_2_ saturation and partial pressure of oxygen ($P_{O_{2}}$) remained unchanged at 98% and 100 mmHg, respectively, thoughout the 1 h heating and cooling protocols (Pearson *et al*. 2011; Chiesa *et al*. 2015).

### Protocol 2: effects of intra‐arterial infusion of ATP on tissue perfusion in normal and heating conditions

Sterile pyrogen‐free ATP (Pharma Waldhof, Dusseldorf, Germany) dissolved in isotonic saline (2 mg ml^−1^; Pharmacy Department, Royal Free Hospital, Hampstead, UK) was infused into the brachial artery at dose rates of 10, 40, 160 and 640 nmol min^−1^ for 4 min at each dose rate, for a total time of 16 min. During the infusion protocol, skin and brachial artery blood flows were measured by laser Doppler flowmetry and Doppler ultrasound, respectively. Blood samples for blood gas variables were taken at each dose rate of ATP. Following 30 min of rest, the arm was heated for 1 h using a water‐perfused cuff with the inlet water temperature of 49°C. The ATP infusion protocol and all measurements were then repeated.

#### Arterial and venous catheterization

To examine the influence of intravascular ATP on limb skin and tissue blood flow, a catheter was inserted into the brachial artery of the non‐dominant arm to infuse ATP and two catheters (18 gauge, 4.5 cm) were inserted in the retrograde direction into an antecubital vein; one for venous blood samples and the other for blood temperature, as described for protocol 1 (*n* = 8). Subjects remained in the supine position throughout, with the arm resting at an 80 deg angle to the torso.

#### Temperature, haemodynamic and blood gases measurements

The *T*_b_, *T*_sk_, brachial artery blood flow, skin blood flow, tissue oxygenation, arterial blood pressure, cardiac output and blood gas data in the ATP infusion protocol were obtained with similar methodology to that reported for protocol 1. In protocol 2, however, *T*_sk_ temperatures were continuously measured using a non‐invasive wearable wireless monitoring system at four different sites on the arm using the iButton remote system (Maxim Integrated, San Jose, CA, USA).

### Protocol 3: effect of heating and cooling on ATP release from blood constituents *in vitro*

The aim of this protocol was to determine how a range of hot and cold temperatures *in vitro* influenced the release of ATP from different blood constituents. Aliquots of erythrocytes, plasma and serum were heated or cooled for 20 min (Baar, 1967) in stirred water‐baths set at 39, 33 (control), 27 and 20°C. Stop solution was added, and samples were immediately centrifuged for 30 s at 15,493*g* before ATP and Hb were determined in the supernatant.

#### Separation of blood constituents for *in vitro* experiments

Blood samples for the *in vitro* heating and cooling protocols were obtained by venepuncture of an antecubital vein from 12 human participants who were tested on the same day of collection (within 30–50 min of blood collection). Blood was collected in a syringe and immediately aliquoted into K3‐EDTA tubes and centrifuged at 15,493*g* for 30 s at room temperature. Plasma was removed and transferred into new tubes; the buffy coat was removed and discarded, whereas the erythrocyte fraction was kept for experimentation. The volume was restored by adding physiological saline after plasma and buffy coat removal from the erythrocyte fraction. Tubes used for serum separation were coated with silicon and micronized silica particles to accelerate clotting (SST II Advance serum separation tubes; Becton & Dickinson, Oxford, Uk). Blood was allowed to clot for 30 min at room temperature, and serum was collected after centrifugation for 10 min at 1000*g*.

### Protocol 4: effect of heating and cooling on ATP release from cultured endothelial cells

To investigate the influence of temperature on ATP release, endothelial cells were seeded onto six‐well plates and grown to confluence. Plates were then removed to water‐baths set to 37 and 39°C or kept at room temperature (23°C). Each well in the six‐well plate represented a different time point of incubation (0, 5, 10, 15, 20 and 30 min) for a particular temperature. One hundred microlitres of supernatant was removed in duplicate, centrifuged and frozen for subsequent ATP analysis. The total number of cells and protein content in each well were assessed after supernatant removal.

#### Culturing endothelial cells

Human umbilical vein endothelial cells (HUVECs) were isolated by incubation with 0.5 mg ml^−1^ collagenase type II in medium 199 (M199; Jaffe *et al*. 1973). Cells were seeded onto 25 cm^2^ plastic culture flasks precoated with 1% gelatine containing M199 supplemented with 20% heat‐inactivated fetal calf serum, 1 mmol l^−1^ sodium pyruvate, antibiotics (150 U ml^−1^ penicillin–150 pg ml^−1^ streptomycin) and 10 ng ml^−1^ heparin‐bound (50 μg ml^−1^) endothelial growth factor. After growing to confluence, cells were subcultured and used at passage 5 or 6. Prior to incubating the cells at different temperatures, cells were carefully washed twice with Hanks’ balanced salt solution (HBSS) and 25 mmol l^−1^ Hepes, pH 7.35. Endothelial cells were allowed to equilibrate for 30 min in a cell culture incubator at 37°C in air enriched with 5% CO_2_ with 1 ml of HBSS and 25 mmol l^−1^ Hepes, pH 7.35 containing the ecto‐ATPase inhibitor ARL67156 (6‐*N*,*N*‐diethyl‐d‐β‐γ‐dibromomethylene adenosine triphosphate; 100 μmol l^−1^, dissolved in Hepes‐buffered HBSS). The temperature in the wells was checked regularly by placing a fine wire thermocouple microprobe in one well, and the temperature was recorded as soon as the plates were placed in the water‐baths. After the 30 min incubation, all cell culture supernatant was removed and cells were detached with the addition of 0.5% trypsin–0.2% EDTA solution. In a small aliquot, the cell density in each well was determined using Trypan Blue staining and counted under a microscope using a haemocytometer. Cells were centrifuged and the pellet was resuspended in a modified radioimmunoprecipitation assay buffer (RIPA) lysis buffer: 50 mmol l^−1^ Tris‐HCl pH 7.4, 150 mmol l^−1^ NaCl, 1 mmol l^−1^ EDTA, 1% Triton X‐100, 0.1% SDS, 1% sodium deoxycholate and 1 mmol l^−1^ phenylmethanesulfonyl fluoride for extraction of protein. Total protein was then determined by the bicinchoninic acid assay. All reagents were purchased from Sigma UK. These experiments were performed on three independent primary HUVEC cultures from different individuals.

### Statistical analysis

To test the effect of heating and cooling over time, the effect of graded ATP infusion in control and heated arm conditions, and the effect of heating and cooling on extracellular ATP in endothelial and blood cells *in vitro*, a two‐way repeated‐measures ANOVA was performed on all dependent variables (brachial artery blood flow, skin blood flow, blood temperature, plasma ATP, etc.) to test for significance. In contrast, we used one‐way repeated‐measures ANOVA to test the effects of ATP infusion in control and heated arm conditions compared with heating alone. When a significant difference (*P* < 0.05) was found, *post hoc* analysis of the data was conducted using Tukey's honestly significant difference test, applying a Bonferroni correction where appropriate. Analyses were performed using SPSS (version 20.0; SPSS Inc., Chicago, IL, USA). Statistical significance was set at *P* < 0.05. Multiple regression for within‐subject repeated measures was used for the analysis of the relationship between blood flow, temperatures and [ATP] (Bland & Altman, 1995). All values are means ± SD.

## Results

### Effect of local arm heating and cooling on blood temperature, haemodynamic and plasma ATP responses *in vivo*

#### Temperatures

**Figure 1:**
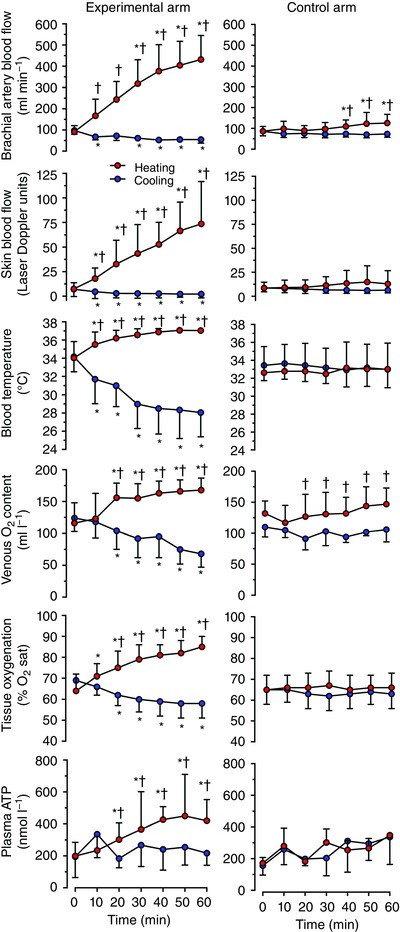
Responses to isolated arm heating and cooling Brachial artery blood flow, forearm skin blood flow, forearm venous blood temperature, venous oxygen content, near‐infrared spectroscopy‐derived tissue oxygenation (expressed as percentage O_2_ saturation; % O_2_ sat) and plasma [ATP] during isolated arm heating or cooling compared with the control arm in healthy individuals resting in the supine position in a thermoneutral environment. Data are shown as means ± SD for 6–10 subjects. ^*^Significantly different from initial 0 min, *P* < 0.05. ^†^Significantly different from cooled experimental arm.

Isolated whole arm heating and cooling over 1 h gradually altered deep venous *T*_b_ (Fig. 1). With heating, *T*_b_ in the experimental arm increased from 34.0 ± 1.8 to 37.0 ± 0.2°C (*P* < 0.05), whereas in the control arm it remained unchanged (32.6–33.0°C). In contrast, cooling decreased *T*_b_ from 34.2 ± 1.6 to 28.1 ± 2.7°C (*P* < 0.05). In the control contralateral arm, *T*_b_ remained stable. In response to arm heating, *T*_sk_ followed a similar pattern, increasing from 29.8 ± 1.4 to 39.5 ± 1.2°C (*P* < 0.05), while remaining unchanged in the control arm at time 0 and after 60 min. With cooling, *T*_sk_ decreased from 29.9 ± 3.5 to 16.3 ± 3.3°C (*P* < 0.05), with no changes in the control arm after 60 min. Core temperature was maintained at ∼37°C throughout each intervention and remained stable until the end of the experimental protocol.

#### Blood flow and systemic haemodynamic responses

**Figure 2:**
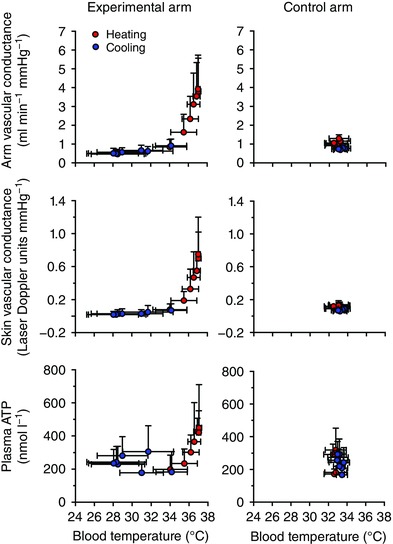
Relationship between blood temperature and arm vascular conductance, skin vascular conductance and plasma [ATP] Arm vascular conductance, skin vascular conductance and venous plasma [ATP] changes with deep venous blood temperature during heating and cooling in the experimental and control arms. Data are shown as means ± SD for 6–10 subjects.

Brachial artery blood flow and forearm skin blood flow gradually increased during the 1 h passive arm heating, becoming elevated by nearly fivefold and by 10‐fold, respectively (Fig. 1). Brachial artery blood flow in the control arm was elevated in the last 10 min of heating, increasing from 86 ± 23 to 126 ± 42 ml min^−1^ at 60 min, respectively (*P* < 0.05). With heating, *V*_mean_ and vessel diameter increased progressively from 12 ± 4 to 46 ± 8 cm s^−1^ (*P* < 0.05) and from 0.41 ± 0.04 to 0.45 ± 0.04 cm (*P* < 0.05), respectively. There was also a small increase in *V*_mean_ in the control unheated arm, which rose to 17 ± 5 cm s^−1^ from baseline (*P* < 0.05), but no increase in brachial artery diameter was observed, and it remained at ∼0.40 cm. Arm vascular conductance increased by fourfold (*P* < 0.05; Fig. 2) after 60 min, in association with the increase in flow. Likewise, skin vascular conductance increased by 10‐fold (*P* < 0.05; Fig. 2) after 60 min. Furthermore, heating augmented venous O_2_ saturation, $P_{O_{2}}$ and O_2_ content in the experimental arm (*P* < 0.05; Table 1). The increase in BABF was accompanied by a decline in the estimated arteriovenous O_2_ difference in both the experimental and control arms from 76 ± 36 to 29 ± 17 ml l^−1^ and from 66 ± 22 to 45 ± 21 ml l^−1^, respectively (*P* < 0.05), but an unchanged estimated forearm ${\dot{V}}_{O_{2}}$ [mean over 1 h protocols: 6 ± 1 and 12 ± 2 ml min^−1^ in the experimental arm for the cooling and heating trials, respectively (environment effect *P =* 0.11; time effect *P =* 0.32) and 7 ± 1 and 6 ± 1 ml min^−1^ for the corresponding controls in the control arm]. The NIRS‐derived tissue oxygenation inclined after the first 10 min of heating rising gradually in the experimental arm (*P* < 0.05; Fig. 1) and remaining unchanged in the control arm. Other measured blood variables, such as [Hb], pH, lactate and osmolality, were not affected by the elevated venous *T*_b_ (Table 1).

**Table 1:** Responses of blood variables to heating and cooling in the experimental and control arms

			Time (min)						
Parameter	Conditions	Arm	0	10	20	30	40	50	60
Haemoglobin (g l^−1^)	Heating	Experimental	144 ± 10	144 ± 9	143 ± 10	141 ± 11	143 ± 8	142 ± 9	143 ± 10
		Control	144 ± 10	145 ± 8	145 ± 12	144 ± 12	143 ± 12	145 ± 11	145 ± 9
	Cooling	Experimental	147 ± 7	146 ± 7	145 ± 7	146 ± 7	148 ± 6	149 ± 7	149 ± 7
		Control	149 ± 5	150 ± 6	150 ± 6	145 ± 13	150 ± 6	150 ± 5	150 ± 7
O_2_ saturation (%)	Heating	Experimental	55 ± 15	61 ± 19*	77 ± 10*†	75 ± 18*†	76 ± 17*†	82 ± 12*†	79 ± 11*†
		Control	66 ± 10	61 ± 15	66 ± 15†	66 ± 17†	67 ± 8†	70 ± 10†	75 ± 10*†
	Cooling	Experimental	60 ± 11	52 ± 13	49 ± 16*	40 ± 13*	40 ± 12*	31 ± 11*	33 ± 11*
		Control	55 ± 8	51 ± 6	47 ± 10*	51 ± 10	45 ± 6*	49 ± 4	49 ± 10
$P_{O_{2}}$ (mmHg)	Heating	Experimental	27 ± 7	35 ± 11†	42 ± 9*†	45 ± 12*†	45 ± 13*†	51 ± 12*†	48 ± 14*†
		Control	33 ± 6†	31 ± 8†	33 ± 9†	34 ± 12†	32 ± 5†	35 ± 8†	38 ± 9*†
	Cooling	Experimental	29 ± 6	23 ± 3*	21 ± 4*	16 ± 3*	16 ± 3*	14 ± 4*	13 ± 4*
		Control	26 ± 3	25 ± 2	23 ± 4	25 ± 3	22 ± 2	24 ± 2	24 ± 3
$P_{CO_{2}}$ (mmHg)	Heating	Experimental	46 ± 5	46 ± 4	42 ± 4	45 ± 5†	45 ± 4†	43 ± 4	44 ± 4
		Control	45 ± 5	46 ± 5	43 ± 6	42 ± 5	42 ± 4	43 ± 5	41 ± 4†
	Cooling	Experimental	45 ± 4	43 ± 6	41 ± 6	39 ± 5*	39 ± 5*	41 ± 5	40 ± 4
		Control	45 ± 5	46 ± 4	46 ± 4	44 ± 6	47 ± 4	46 ± 5	46 ± 5
pH	Heating	Experimental	7.41 ± 0.02	7.41 ± 0.02	7.41 ± 0.03	7.40 ± 0.03	7.40 ± 0.02	7.40 ± 0.02	7.40 ± 0.02
		Control	7.40 ± 0.04	7.40 ± 0.03	7.44 ± 0.06	7.41 ± 0.03	7.40 ± 0.03	7.40 ± 0.03	7.42 ± 0.03
	Cooling	Experimental	7.41 ± 0.03	7.44 ± 0.04	7.44 ± 0.04	7.47 ± 0.05	7.47 ± 0.05	7.45 ± 0.04	7.44 ± 0.03
		Control	7.41 ± 0.03	7.40 ± 0.03	7.40 ± 0.03	7.40 ± 0.03	7.40 ± 0.03	7.40 ± 0.03	7.40 ± 0.04
Lactate (mmol l^−1^)	Heating	Experimental	1.1 ± 0.3	1.0 ± 0.3		0.9 ± 0.4	0.8 ± 0.2	0.8 ± 0.3	0.8 ± 0.3
		Control	1.3 ± 0.3	1.2 ± 0.3	1.1 ± 0.3	0.9 ± 0.5	1.0 ± 0.3	1.0 ± 0.2	1.0 ± 0.4
	Cooling	Experimental	1.1 ± 0.3	1.2 ± 0.3	1.1 ± 0.2	1.1 ± 0.3	1.1 ± 0.2	1.1 ± 0.2	1.1 ± 0.2
		Control	1.3 ± 0.2	1.3 ± 0.2	1.3 ± 0.1	1.3 ± 0.3	1.3 ± 0.3	1.2 ± 0.3	1.3 ± 0.3
Osmolality	Heating	Experimental	280 ± 3	279 ± 5	280 ± 5	280 ± 4	279 ± 5	279 ± 5	279 ± 5
(mosmol kg^−1^)		Control	281 ± 3	280 ± 5	281 ± 5	281 ± 5	282 ± 4	281 ± 4	281 ± 4
	Cooling	Experimental	280 ± 4	280 ± 4	281 ± 5	280 ± 4	280 ± 4	280 ± 3	279 ± 4
		Control	282 ± 3	282 ± 3	282 ± 3	283 ± 4	282 ± 3	282 ± 3	281 ± 3

Data are shown as means ± SD for eight subjects. Abbreviations: $P_{CO_{2}}$, partial pressure of carbon dioxide; and $P_{O_{2}}$, partial pressure of oxygen. Note that pH, $P_{O_{2}}$ and $P_{CO_{2}}$ were corrected for blood temperature. ^*^Different from baseline (0), *P* < 0.05. ^†^Different from respective cooling, *P* < 0.05.

In contrast to heating, localized cooling of the experimental arm steadily decreased BABF by 44% (*P* < 0.05) at 60 min. Here, *V*_mean_ was reduced from 12 ± 6 to 7 ± 3 cm s^−1^ (*P* < 0.05); however, there were no changes in brachial artery diameter (0.41 ± 0.05 *versus* 0.41 ± 0.04 cm). Blood flow in the control non‐cooled arm remained unchanged (Fig. 1). Similar patterns for both the experimental and the control arm were observed for forearm skin blood flow. Decreased flow with cooling was accompanied by an increase in the arteriovenous O_2_ difference in the experimental arm (*P* < 0.05; Table 1). Arm vascular conductance with cooling decreased by nearly half, with a slight decrease in the control non‐cooled arm (Fig. 2). Skin vascular conductance decreased by nearly fourfold with cooling (Fig. 2). Venous O_2_ saturation and $P_{O_{2}}$ in the experimental arm decreased with cooling (*P* < 0.05; Table 1). Tissue oxygenation followed a similar pattern, decreasing (*P* < 0.05) in the experimental arm and remaining stable in the control arm after 60 min. Cooling did not change [Hb], pH, lactate or plasma osmolality in either the experimental or the control arm (Table 1).

Systemic responses with passive arm heating were unchanged during the first 40 min, but a small increase in cardiac output and mean arterial pressure were observed from 50 to 60 min (Table 2). Overall, the combined representation of *T*_b_ changes with either arm or skin vascular conductance demonstrated a strong association, with coefficient of determination of *r* ^2^ = 0.50 and *r* ^2^ = 0.49, respectively (both *P* < 0.05; Fig. 2). These responses suggest that an increase in conductance with heating indicates vasodilatation and a decrease in conductance with cooling, vasoconstriction. In contrast, no association between temperature and vascular conductance was observed in the control arm.

**Table 2:** **Systemic responses during arm heating and cooling**

		Time (min)						
Parameter	Conditions	0	10	20	30	40	50	60
Cardiac output (l min^−1^)	Heating	5.7 ± 1.7	5.9 ± 1.8	5.9 ± 1.5	5.7 ± 1.6	5.8 ± 1.8	6.0 ± 2.1*	6.3 ± 1.5*
	Cooling	5.8 ± 1.4	5.7 ± 1.3	5.9 ± 1.8	5.9 ± 1.7	5.9 ± 1.7	6.1 ± 1.2	5.8 ± 2.1
Heart rate (beats min^−1^)	Heating	62 ± 8	64 ± 11	64 ± 12	62 ± 9	63 ± 9	63 ± 9	65 ± 8
	Cooling	63 ± 9	60 ± 7*	60 ± 8*	61 ± 9	61 ± 9	61 ± 8	62 ± 9
Stroke volume (ml)	Heating	91 ± 22	92 ± 20	91 ± 19	91 ± 18	90 ± 17	93 ± 15	94 ± 15
	Cooling	91 ± 20	93 ± 18	96 ± 14	96 ± 19	96 ± 18	98 ± 14	95 ± 26
Mean arterial pressure (mmHg)	Heating	93 ± 13	93 ± 8	95 ± 12	92 ± 13	95 ± 12	95 ± 12	98 ± 8*
	Cooling	97 ± 13	96 ± 16	98 ± 13	99 ± 13	101 ± 13	101 ± 13	100 ± 8
Systolic blood pressure (mmHg)	Heating	126 ± 17	127 ± 17	127 ± 17	123 ± 17	129 ± 17	129 ± 16	133 ± 14*
	Cooling	133 ± 14	131 ± 19	137 ± 17	137 ± 17	137 ± 18	140 ± 18*	145 ± 18*
Diastolic blood pressure (mmHg)	Heating	71 ± 17	72 ± 13	72 ± 14	72 ± 17	74 ± 15	73 ± 13	76 ± 13*
	Cooling	75 ± 10	75 ± 12	77 ± 10	76 ± 10	78 ± 12	79 ± 10	82 ± 11*
Systemic vascular conductance	Heating	63 ± 21	64 ± 19	63 ± 18	64 ± 20	61 ± 15	64 ± 22	66 ± 20
(ml min^−1^ mmHg^−1^)	Cooling	59 ± 12	59 ± 11	60 ± 13	60 ± 15	60 ± 14	60 ± 13	59 ± 15

Data are shown as means ± SD for eight subjects. ^*^Different from baseline (0), *P* < 0.05.

#### Circulating plasma ATP responses

Prior to heating and cooling, deep venous plasma [ATP] was not different at rest in either arm (199 ± 86 *versus* 172 ± 31 nmol l^−1^ in the experimental and control arms). As the arm was heated, [ATP] gradually doubled after 1 h (*P* < 0.05; Fig. 1), whereas in the control unheated arm [ATP] remained stable. Cooling the experimental arm for 1 h did not change [ATP]. The association of *T*_b_ with plasma [ATP] shows that the relationship is strong during the heating trial (*r* ^2^ = 0.33, *P =* 0.036), whereas during cooling the relationship was absent (*r* ^2^ = 0.00, *P =* 0.58; Fig. 2). In addition, the relationship between arm vascular conductance and deep venous plasma [ATP] during heating was highly significant (*r* ^2^ = 0.49, *P =* 0.002).

### Effects of intra‐arterial infusion of ATP on local skin and arm perfusion in normal and heating conditions

**Figure 3:**
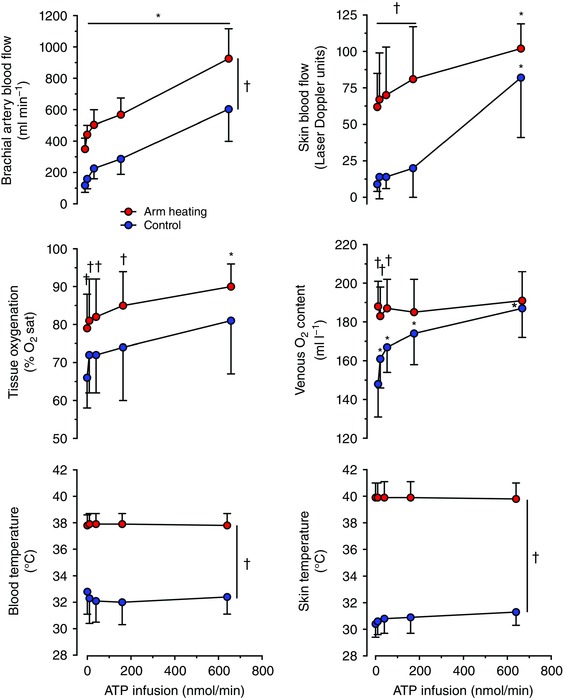
Responses to intrabrachial artery infusion of ATP Brachial artery blood flow, near‐infrared spectroscopy‐derived tissue oxygenation (expressed as percentage O_2_ saturation; % O_2_ sat), forearm superficial venous blood temperature, forearm skin blood flow, forearm venous blood oxygen content and forearm skin temperature at baseline (0) and during intrabrachial artery ATP infusion in control and heated‐arm conditions in healthy individuals resting in the supine position in a thermoneutral environment. Data are shown as mean ± SD for six to eight male participants. ^*^Significantly different from baseline (0), *P* < 0.05. ^†^Significantly different from the control conditions.

During ATP infusion in normal conditions, *T*_b_ and skin temperature remained stable at ∼33 and ∼31°C, respectively, throughout the infusion protocol (Fig. 3). After heating for 1 h, *T*_b_ and skin temperature reached 38 and 40°C, respectively, remaining at this level throughout (Fig. 3). In contrast, BABF increased significantly with ATP infusion compared with baseline, both in control conditions (603 ± 205 *versus* 118 ± 45 ml min^−1^) and during arm heating (926 ± 191 *versus* 350 ± 70 ml min^−1^; Fig. 3). Forearm skin blood flow during control conditions increased from baseline only with the highest ATP infusion dose, rising by ninefold (*P* < 0.05). Forearm skin blood flow was 62 ± 23 laser Doppler units after heating and again rose significantly with the highest dose to 102 ± 17 laser Doppler units (*P* < 0.05). The change (Δ) in BABF between the highest ATP dose and baseline was similar in control and heating conditions (∆ 485 ± 205 *versus* 577 ± 168 ml min^−1^, respectively; *P* > 0.05), but these vasodilator effects of ATP infusion were approximately twofold greater (*P* < 0.03) than the effects of isolated arm heating (∆ 232 ± 54 ml min^−1^; *P* < 0.03). In contrast, the change in skin blood flow was similar in the three conditions (∆ 72 ± 39, 52 ± 21 and 41 ± 24 laser Doppler units with ATP infusion alone, isolated arm heating and ATP infusion in the heated arm, respectively; *P =* 0.275).

The increased BABF with ATP infusion in control conditions was reflected by increases in venous O_2_ saturation, $P_{O_{2}}$ and O_2_ content (*P* < 0.05; Table 3 and Fig. 3). However, increases in BABF with ATP infusion after heating were not associated with significant alterations in venous O_2_ saturation, $P_{O_{2}}$ and O_2_ content (Table 3). The NIRS‐derived tissue oxygenation increased with ATP infusion compared with baseline during arm heating (90 ± 6 *versus* 79 ± 9%; *P* < 0.05). In the control trial, the estimated forearm arteriovenous O_2_ difference declined from 45 ± 2 ml l^−1^ at baseline to 4 ± 4 ml l^−1^ with the highest dose of ATP infusion, but the combination of ATP infusion and arm heating did not alter this response (3 ± 2 ml l^−1^). However, the corresponding estimated forearm ${\dot{V}}_{O_{2}}$ remained unchanged across conditions inducing an ∼800 ml min^−1^ increase in BABF above baseline (forearm ${\dot{V}}_{O_{2}}$ grant mean 4 ± 1 ml min^−1^; effect of ATP infusion *P =* 0.56; effect of environment *P =* 0.11; interaction *P =* 0.40). With regard to systemic haemodynamic responses, the 640 nmol min^−1^ ATP infusion after arm heating induced a significant increase in cardiac output, but not in the control conditions (Table 4).

**Table 3:** Responses of blood variables during intrabrachial artery ATP infusion in control and heated‐arm conditions

		Intrabrachial artery ATP infusion (nmol min^−1^)				
Parameter	Conditions	0	10	40	160	640
Haemoglobin (g l^−1^)	Control	143 ± 10	143 ± 10	142 ± 9	142 ± 9	141 ± 10
	Heat stress	145 ± 11	143 ± 11	143 ± 11	143 ± 11	143 ± 11
O_2_ saturation (%)	Control	74 ± 6	83 ± 6	86 ± 5	89 ± 6*	96 ± 2*
	Heat stress	94 ± 2†	94 ± 2†	95 ± 2†	95 ± 2†	97 ± 1
$P_{O_{2}}$ (mmHg)	Control	32 ± 5	37 ± 7	40 ± 6	46 ± 8	59 ± 9*
	Heat stress	73 ± 6†	72 ± 9†	78 ± 7†	78 ± 11†	87 ± 7†
$C_{vO_{2}}$ (ml l^−1^)	Control	148 ± 17	161 ± 15	167 ± 13	174 ± 16	187 ± 15*
	Heat stress	188 ± 13†	183 ± 15†	187 ± 15†	185 ± 17	191 ± 15
$P_{CO_{2}}$ (mmHg)	Control	38 ± 5	37 ± 4	36 ± 4	34 ± 3	34 ± 6*
	Heat stress	41 ± 3†	40 ± 4†	40 ± 5†	39 ± 5†	39 ± 4†
pH	Control	7.42 ± 0.03	7.42 ± 0.02	7.44 ± 0.02	7.45 ± 0.02	7.45 ± 0.01
	Heat stress	7.37 ± 0.02†	7.38 ± 0.01†	7.38 ± 0.01†	7.38 ± 0.01†	7.39 ± 0.01*†
Lactate (mmol l^−1^)	Control	1.1 ± 0.4	1.0 ± 0.4	1.0 ± 0.4	0.8 ± 0.3	0.9 ± 0.3
	Heat stress	0.8 ± 0.2†	0.7 ± 0.3†	0.7 ± 0.2†	0.7 ± 0.2†	0.7 ± 0.2†
Osmolality (mosmol kg^−1^)	Control	290 ± 7	291 ± 8	287 ± 5	288 ± 7	286 ± 6
	Heat stress	292 ± 6	288 ± 5*	284 ± 5*†	285 ± 4*†	284 ± 4*

Data are shown as means ± SD for eight subjects. Abbreviations: $C_{vO_{2}}$, venous oxygen content; $P_{CO_{2}}$, partial pressure of carbon dioxide; and $P_{O_{2}}$, partial pressure of oxygen. Note that pH, $P_{O_{2}}$ and $P_{CO_{2}}$ were corrected for blood temperature. ^*^Different from baseline (0), *P* < 0.05. ^†^Different from control, *P* < 0.05.

**Table 4:** Systemic responses during ATP infusion

		Intrabrachial artery ATP infusion (nmol min^−1^)				
Parameter	Conditions	0	10	40	160	640
Cardiac output (l min^−1^)	Control	5.4 ± 0.7	5.3 ± 0.6	5.4 ± 0.8	5.4 ± 0.8	5.7 ± 0.6
	Heat stress	5.5 ± 0.9	5.6 ± 0.9	5.7 ± 1.2	5.8 ± 0.9	6.2 ± 1.0*
Heart rate (beats min^−1^)	Control	56 ± 8	54 ± 8	54 ± 7	54 ± 9	56 ± 9
	Heat stress	58 ± 8	58 ± 11	58 ± 11	59 ± 9	60 ± 8
Stroke volume (ml)	Control	98 ± 21	101 ± 22	101 ± 20	102 ± 21	103 ± 20
	Heat stress	98 ± 18	98 ± 20	100 ± 20	100 ± 20	102 ± 20
Mean arterial pressure (mmHg)	Control	88 ± 4	90 ± 6	90 ± 8	89 ± 8	90 ± 6
	Heat stress	88 ± 4	88 ± 4	88 ± 6	90 ± 5	89 ± 5
Systolic blood pressure (mmHg)	Control	129 ± 5	134 ± 7	133 ± 8	133 ± 7	135 ± 4
	Heat stress	126 ± 7	126 ± 6	128 ± 12	131 ± 9	129 ± 9
Diastolic blood pressure (mmHg)	Control	68 ± 5	69 ± 6	69 ± 9	68 ± 8	69 ± 7
	Heat stress	68 ± 4	68 ± 4	68 ± 5	70 ± 4	68 ± 6
Systemic vascular conductance	Control	62 ± 10	60 ± 10	61 ± 14	61 ± 13	63 ± 10
(ml min^−1^ mmHg^−1^)	Heat stress	63 ± 12	63 ± 12	63± 13	64 ± 11	70 ± 12

Data are shown as means ± SD for seven or eight subjects. ^*^Different from baseline (0), *P* < 0.05.

### Effects of heating and cooling on ATP release from blood constituents *in vitro*

**Figure 4:**
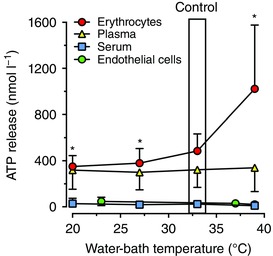
*In vitro* release of ATP with heating and cooling Erythrocyte, plasma and serum samples were incubated in water‐baths set at 39, 33 (control), 27 and 20°C. Release of ATP was measured in blood samples taken from 12 subjects. ^*^Significantly different from 33°C (control). Data are shown as means ± SD. Separately, confluent cultures of human umbilical vein endothelial cells (HUVECs) were incubated in water‐baths set at 37 (control), 39 and 23°C (room temperature) for 30 min. Release of ATP was measured in duplicate from three independent primary HUVEC cultures from different individuals. Data are shown as means ± SD.

To investigate the effects of *in vitro* heating and cooling on the release of ATP from isolated erythrocytes, plasma and serum, samples were exposed to physiological temperatures in water‐baths set at 39, 33 (control), 27 and 20°C for 20 min. The ATP release from isolated erythrocyte was nearly doubled from 33 to 39°C (*P* < 0.05; Fig. 4). ATP release from erythrocytes exposed to 27 and 20°C was reduced to 380 ± 127 and 350 ± 95 nmol l^−1^ (*P* < 0.05) compared with the control (33°C). In contrast, no changes in ATP concentrations were observed in either isolated plasma or serum samples incubated at these temperatures.

### Effects of heating and cooling on ATP release from human endothelial cells

In cell supernatants from confluent cultures of HUVECs incubated for 30 min in an incubator set at standard cell culture conditions of 37°C and air supplemented with 5% CO_2_, the extracellular [ATP] was 49 ± 24 nmol l^−1^. The cells were then moved to water‐baths after this equilibration time. The release of extracellular ATP over 30 min incubation showed a trend to decline at both 37 and 39°C to 29 ± 21 and 20 ± 15 nmol l^−1^, whereas the ATP concentration was preserved at 23°C (46 ± 36 nmol l^−1^; *P =* 0.085; Fig. 4). To account for any loss of cells in the supernatant as a result of the change in temperature, adherent cells were detached and counted. The initial average cell count was 11 ± 5 × 10^4^ cells ml^−1^, and after 30 min there were 7 ± 3, 9 ± 3 and 10 ± 6 × 10^4^ cells ml^−1^ at 39 and 23 compared with 37°C, respectively (*P =* 0.13). The ATP in the supernatant normalized to total protein was initially 1.4 ± 1.0 nmol l^−1^ (mg protein)^−1^, and 30 min incubation at 39 and 23 compared with 37°C resulted in no differences [0.8 ± 0.4, 1.1 ± 0.8 and 0.6 ± 0.4 nmol l^−1^ (mg protein)^−1^, respectively; *P =* 0.072].

## Discussion

There were five novel findings in this study. Firstly, forearm perfusion and vascular conductance both increased progressively with elevations in *T*_b_ and decreased with graded reductions in *T*_b_, indicating a close coupling between local tissue blood flow and *T*_b_ variations with local heating and cooling. The alteration in perfusion was paralleled by an inverse response in forearm O_2_ extraction due to modifications in venous O_2_ content, such that ${\dot{V}}_{O_{2}}$ did not change. Secondly, the *in vivo* plasma [ATP] was correlated closely with increasing *T*_b_ with heating. However, this relationship was not apparent with cooling. Thirdly, intra‐arterial infusion of ATP increased skin and deep forearm tissue blood flow and vascular conductance both in control conditions and after passive heating, independently of *T*_b_, skin and core temperatures. Fourthly, in isolated erythrocytes *in vitro*, heating and cooling significantly altered the concentration of ATP compared with incubation of erythrocytes at the control temperature. Fifthly, exposure of human endothelial cells to both heating and cooling did not stimulate a change in the concentration of extracellular ATP. Collectively, our findings suggest that the erythrocytes are involved in thermal regulation of tissue blood flow via modulation of ATP release.

### Limb tissue perfusion and plasma ATP with local heating, cooling and ATP infusion

In the present study, brachial artery blood flow increased gradually in response to local heating (105 ± 25 ml min^−1^ °C^−1^) and decreased progressively with cooling, but at a significantly lower rate (7 ± 1 ml min^−1^ °C^−1^). These distinct forearm blood flow responses to local heating and cooling occurred essentially without alterations in core temperature, haematological variables or blood osmolality, pH, lactate or electrolytes (Tables 1 and 2). Moreover, the alterations in forearm perfusion were paralleled by inverse responses in arteriovenous O_2_ differences and NIRS‐derived tissue oxygenation but an unchanged local tissue metabolism as judged by the similar forearm ${\dot{V}}_{O_{2}}$ in the heating and cooling conditions ($\Delta {\dot{V}}_{O_{2}}$ = 5 ± 3 ml min^−1^; *P =* 0.11). This non‐significant local metabolic response is in contrast to what generally occurs during hand‐grip exercise, where comparable forearm hyperaemia in young healthy individuals is met by a 50 ± 5 ml min^−1^ increase in forearm ${\dot{V}}_{O_{2}}$ (Richards *et al*. 2015). These observations collectively suggest that the aforementioned variables and/or metabolically derived signals did not account for the ∼400 ml min^−1^ difference in forearm blood flow between conditions (i.e. sevenfold difference).

The magnitude of the increase in BABF during the heating protocol was similar to the values observed in studies comparing the effects of local forearm heating (Padilla *et al*. 2011) and whole‐body passive heating (Barcroft *et al*. 1947; Ooue *et al*. 2007). A common factor in the whole‐body heating studies is that the internal temperature was increased significantly. Conversely, exposure to direct ice cooling in the present study resulted in a 44% reduction in brachial artery blood flow, which is in general agreement with results from classic studies (Barcroft & Edholm, 1943; Karunakara *et al*. 1999) and more recent work reporting reductions in brachial artery blood flow with cold water immersion (Yanagisawa *et al*. 2007) and during repeated cold pressor tests (Ade *et al*. 2012). However, these experimental cooling interventions are known to increase metabolism, mean arterial pressure and tissue perfusion pressure, which would influence the degree of vasoconstriction induced by cold exposure. Taken as a whole, the present experimental design minimized the potential confounding metabolic and reflex responses (as confirmed by the virtual lack of change in tissue perfusion and oxygenation in the control arm) associated with interventions such as whole‐body heat and cold stress and following exercise hyperaemia (Mourot *et al*. 2009; Pearson *et al*. 2011).

A pivotal aim of the study was to gain insight into vascular thermosensitive mechanisms of local blood flow control during thermal interventions and/or therapies. In the presence of an unchanged forearm perfusion pressure, blood flow and vascular conductance changed in parallel in the present study. This indicates that the increases in forearm tissue blood flow with heating were evoked by net vasodilatation, whereas the decreases in tissue blood flow with cooling were attributable to net vasoconstriction. The question then arises: what mechanisms underpin these contrasting local haemodynamic responses? Regulation of local tissue perfusion is thought to be the result of the interaction between neural sympathetic activity and locally derived vasoactive substances. Muscle and skin sympathetic activity may increase with both heating and cooling (Normell & Wallin, 1974; Niimi *et al*. 1997; Cui *et al*. 2007). However, the increased sympathetic nerve activity can be modulated by vasodilator signals sensitive to increases and decreases in temperature such that vasodilatation prevails with heating and the rate of vasoconstriction with cooling is blunted.

A novel aspect of the present study, which made it possible to investigate potential temperature‐related intravascular mechanisms *in vivo*, was the continuous measurement of *T*_b_ in an antecubital deep vein coupled with the frequent measurements of plasma ATP and blood gases from the same vessel and regular or continuous measures of blood flow and tissue oxygenation. A salient finding of the present study is the intimate relationship between changes in forearm vascular conductance and variations in deep venous *T*_b_ with local heating (*r* ^2^ = 0.50; *P =* 0.035), which was paralleled by the same relationship between skin vascular conductance and changes in *T*_b_ (*r* ^2^ = 0.49; *P =* 0.001). This observation supports the possibilities that temperature *per se* and/or temperature‐sensitive vascular mechanisms contribute to the regulation of local tissue blood flow in conditions that increase blood and tissue temperature. In the present study, deep venous *T*_b_ rose from 34 to 37°C with heating and fell to 28°C with cooling. Although we did not measure muscle temperature, similar studies using thermocouples placed 3 cm into the brachioradialis muscle found that external heating and cooling to 45, 32 and 17°C changed muscle temperature from 33°C to 38.6, 35.8 and 28.3°C, respectively (Abramson *et al*. 1959; Detry *et al*. 1972; Pearson *et al*. 2011). It is therefore likely that blood and deep tissue temperature in the forearm varied by as much as 9–10°C after 1 h of heating and cooling. Interestingly, the studies assessing the impact of temperature variations in*in vitro* skeletal muscle vessel preparations show, for the most part, that temperature *per se* does not exert a direct effect on smooth muscle contractile function (Vanhoutte & Shepherd, 1970; Ives *et al*. 2011). This lends support to the alternative hypothesis that temperature is predominantly acting indirectly on the limb tissue vasculature via temperature‐sensitive vascular mechanisms.

### Mechanism of temperature‐dependent regulation of skin and tissue perfusion

One of the potential intravascular mechanisms contributing to the regulation of limb tissue blood flow with heating and cooling could be the recently proposed temperature‐dependent release of ATP from circulating erythrocytes (Kalsi & González‐Alonso, 2012). A novel observation in support of this mechanism are the intimate relationships between the increases in brachial artery blood flow (fivefold) and the increases in deep venous plasma [ATP] (*r* ^2^ = 0.45; *P =* 0.004) and the rise in skin blood flow (10‐fold) and elevations in plasma [ATP] (*r* ^2^ = 0.46; *P* < 0.01) seen with isolated arm heating in the present study. This finding expands previous observations from our laboratory documenting a close relationship between increases in leg tissue blood flow, arterial plasma [ATP] and muscle temperature during whole‐body heating and repeated exercise (Pearson *et al*. 2011) and provides strong evidence that during direct local heat stress, local *T*_b_ rather than core temperature stimulates the increase in plasma [ATP]. This in turn causes vasodilatation by binding to P_2Y_ purinergic receptors in the vascular endothelium and smooth muscle and stimulating the release of NO and prostaglandins (Mortensen *et al*. 2009 *a*) and/or activating inward rectifying potassium channels (Crecelius *et al*. 2012).

Importantly, in the present study deep venous blood O_2_ content increased by up to 70% with heating, indicating that augmented temperature‐stimulated erythrocyte ATP release is unrelated to the well‐characterized mechanism sensitive to reductions in blood oxygenation that occur during exercise (Ellsworth *et al*. 1995; Jagger *et al*. 2001; Sprague *et al*. 2009). Increases in temperature are widely known to favour the offloading of O_2_ in metabolically active tissues by reducing the affinity of haemoglobin for O_2_ as the oxyhaemoglobin dissociation curve is shifted to the right in *in vitro* conditions (Barcroft & King, 1909; Duc & Engel, 1969). This phenomenon, which is associated with augmented erythrocyte ATP release during exercise (Ellsworth *et al*. 1995; González‐Alonso *et al*. 2002, 2015), is overridden during passive heating *in vivo* because venous O_2_ saturation and tension increase drastically when O_2_ delivery rises in the face of an unchanged metabolic demand. In contrast to the responses to heating, plasma [ATP] did not change with graded cooling *in vivo*. This finding might seem surprising in light of the parallel *in vitro* observation that ATP release from erythrocytes decreased with decreasing blood temperatures (Fig. 4). The argument that a lower intravascular ATP is associated with reduced forearm blood flow has been made in the literature when associating the reductions in exercise hyperaemia with ageing in humans with blunted plasma ATP (Kirby *et al*. 2012). However, there are at least two potential explanations for the unchanged plasma [ATP] with cooling *in vivo* in the present study. Firstly, arm cooling led to an increased tissue oxygen extraction secondary to reduced tissue perfusion. Conversely to our finding with heating, decreases in oxyhaemoglobin and $P_{O_{2}}$ have repeatedly been shown to be strong stimuli for erythrocyte ATP release (Jagger *et al*. 2001; Sprague *et al*. 2009). Therefore, it is possible that erythrocyte ATP release remained activated by the lowering haemoglobin oxygenation and $P_{O_{2}}$ despite a diminishing temperature stimulus and contributed to the lower rate of decrease in blood flow per degree in temperature with cooling *versus* heating. Secondly, the lower *T*_b_ might have attenuated ATP degradation activity, as turnover of ATP degradation and formation is slowed down at lower temperatures (Mohr *et al*. 2010). Irrespective of the mechanism underlying the unchanged plasma [ATP] with cooling *in vivo*, the present results provide partial support for the first hypothesis of the study, because the increases in forearm blood flow with heating are closely related to elevations in both *T*_b_ and plasma [ATP], but the smaller reductions in forearm perfusion with cooling are associated only with the reduction in *T*_b_.

Another important finding is that physiological variations in temperature ranging from 20 to 39°C influence ATP release from isolated human erythrocytes, but do not affect ATP release from plasma, serum or endothelial cells. We have previously shown in *in vitro* experiments that heating plasma or serum from 33 to 42°C also does not increase the release of ATP (Kalsi & González‐Alonso, 2012). Expanding this temperature range, we now show an unchanged ATP release from plasma and serum when the temperature is lowered to 27 and 20°C (Fig. 4). In contrast, erythrocyte ATP release *in vitro* doubled with heating at 39°C compared with the control at 33°C, whereas a significant reduction in erythrocyte ATP release was observed with cooling to 27 and 20°C. The idea that haemoglobin molecules in the RBCs are temperature ‘sensors’ has been studied by others by measuring the temperature‐sensitive changes occurring in the lipid bilayers and Hb protein unfolding in RBCs (Artmann *et al*. 2009; Stadler *et al*. 2012). Studies on endotherms and ectotherms found that membrane fluidity and partial unfolding of Hb were different depending on body temperature, which might also have an influence on the release of ATP from erythrocytes through protein transporters. Endothelial cells could be another source of intravascular ATP *in vivo*, at least in response to stimuli such as shear stress and hypoxia (Bodin *et al*. 1992; Bodin & Burnstock, 1995), whereas others have shown an attenuation (Faigle *et al*. 2008). Surprisingly, our finding that the concentration of extracellular ATP was unchanged in human endothelial cells exposed to different temperatures suggests that endothelial cells may not have the necessary temperature‐sensitive mechanisms to release ATP or these cells display very high NTPDase1/CD39 and other ectonucleotidases, and their activities are activated during increasing temperature. Therefore, based on unchanged extracellular ATP concentrations in cultured HUVECs incubated at different temperatures, net ATP balance/homeostasis remains unchanged (i.e. it is still possible that ATP is released more efficiently at higher temperatures, but it is compensated by rapid degradation through concurrently activated NTPDase activity; Yegutkin *et al*. 2012). Although temperature‐sensitive transient receptor potential (TRP) channel mechanisms have been described in corneal endothelial cells (Mergler *et al*. 2010), ATP release from these channels has not been demonstrated. Taken together, the findings from the present and previous *in vitro* experiments (Kalsi & González‐Alonso, 2012) indicating that erythrocyte ATP release is sensitive to physiological increases and decreases in blood temperature support the hypothesis that the erythrocytes might function as thermal sensors and ATP signalling generators for control of tissue perfusion during thermal interventions.

Arterial ATP infusion has long been known to increase human limb blood flow (Rongen *et al*. 1994; González‐Alonso *et al*. 2002; Crecelius *et al*. 2012). However, the role of intravascular ATP as a thermal mediator of skin and limb deep tissue vasodilatation has not been investigated. To gain insight into this mechanism, we infused ATP at four doses to increase intravascular ATP availability gradually in normal and local hyperthermic conditions, in which *T*_b_ and skin temperature were clamped at either ∼32–33 or ∼38–40°C, respectively (Fig. 3). An immediate increase in BABF and venous O_2_ content was observed with even the lowest ATP dose (10 nmol min^−1^) in both control and hyperthermic conditions, whereas a significant increase in skin blood flow in both conditions was apparent only during the highest infusion rate (640 nmol min^−1^, comparable to the observed erythrocyte ATP at high temperature *in vitro*). These observations show that ATP infusion in a conduit artery leads to increases in perfusion to muscle and other deep tissues across a range of infusion rates and concomitant elevations in blood and tissue oxygenation, but increases skin blood flow only at high ATP infusion doses. An explanation for this could be that the availability of ATP in the skin vessels remains unaltered when ATP is infused at low doses because it is used as it travels from the deep tissue circulation to the skin and only at high doses reaches the skin vasculature and causes vasodilatation. Interestingly, this skin blood flow response is similar to the one resulting from direct administration of ATP into the cutaneous interstitial space by intradermal microdialysis, where an increase in skin perfusion was observed only above an infusion rate of 120 nmol min^−1^ (Fujii *et al*. 2015). This observation, together with the evidence that local heating does not result in an accumulation of ATP in the interstitial space of human skin (Gifford *et al*. 2012) and that ATP infusion does not alter muscle interstitial ATP (Mortensen *et al*. 2009 *b*), point to a dominant role of intravascular rather than interstitial mechanisms in thermal and ATP‐induced hyperaemia. In this light, the administration of a low dose of ATP to the muscle interstitium does not alter tissue blood flow in human limbs and even causes vasoconstriction of the rat gluteus maximus muscle microcirculation (Nyberg *et al*. 2013). This contrasts to the increases in limb deep tissue flow seen in the present study when the same dose of ATP was administered intraluminally (Fig. 3). Studies infusing the degradation metabolite adenosine have shown that vasodilatation occurs in different limb tissues, including muscle, skin, fat and bone (Rådegran & Calbet, 2001; Heinonen *et al*. 2010). This raises the possibility that ATP is rapidly degraded by ectonucleotidases (Zimmermann, 1996) and causes vasodilatation via adenosine activation of P_1_ receptors, rather than a direct effect of ATP on P_2Y_ receptors. However, the finding that blockade of adenosine receptors with theophylline does not alter ATP‐induced limb vasodilatation in the human forearm or leg (Rongen *et al*. 1994; Mortensen *et al*. 2009 *b*) suggests that this is an unlikely possibility. ATP, but not its degradation compounds ADP, AMP or adenosine, can also override the increases in α‐adrenergic vasoconstrictor activity evoked by tyramine‐induced release of noradrenaline, and has a much greater vasodilator potency than its dephosphorylated metabolites (Rosenmeier *et al*. 2008). Interestingly, when skin and deep tissue blood flow was already high as a result of local hyperthermia, ATP infusion led to the same limb tissue hyperaemia (∼0.5 l min^−1^) but about one‐half of the skin hyperaemia seen in control conditions. This is suggestive of a differential ATP availability and/or purinergic receptor sensitivity of deep tissue and skin to exogenous ATP administration (Kluess *et al*. 2005). Although studies blocking the downstream purinergic receptors in different thermal conditions and ATP infusions are warranted to establish a cause‐and‐effect relationship, the present and previous findings collectively suggest that the temperature‐mediated elevation in erythrocyte ATP release has the capacity to activate endothelial purinergic signalling and downstream endothelial and vascular smooth muscle vasodilatory pathways and increase in limb tissue and skin blood flow to levels above those seen during local heating *in vivo* in humans.

In summary, our findings indicate that increases in human limb skin and deep tissue blood flow with local heating are closely associated with temperature‐mediated elevations in ATP release from erythrocytes; however, the smaller reductions in tissue perfusion with cooling are unrelated to a reduciton in intravascular ATP *in vivo*. Increasing intravascular ATP availability with ATP infusion increases skin blood flow to levels equal to thermally induced cutaneous hyperaemia. The present study supports the hypothesis that the thermosensitive erythrocyte‐derived ATP signalling mechanism contributes to the control of local skin and deep tissue perfusion and provides evidence that local heating and cooling are methods of manipulating the supply of regulatory substances, oxygen and nutrients to human tissues for therapeutic and ergogenic benefit.

## Additional information

### Competing interests

None declared.

### Author contributions

Experiments were performed at the Centre for Human Performance, Exercise and Rehabilitation, Brunel University London. K.K.K. and J.G.‐A. conceived and designed the studies. L.A. and M.D.L. provided clinical support for the ATP infusion protocol. All other authors were involved in data collection, analysis and interpretation of data. K.K.K. drafted the article, and it was critically revised for important intellectual content by S.T.C., S.J.T. and J.G.‐A. All authors approved the final version of the manuscript and agree to be accountable for all aspects of the work in ensuring that questions related to the accuracy or integrity of any part of the work are appropriately investigated and resolved. All persons designated as authors qualify for authorship, and all those who qualify for authorship are listed.

### Funding

This research received no specific grant from any funding agency in the public, commercial or not‐for‐profit sectors.
